# Dengue with Hemorrhagic Manifestations and Acute Pancreatitis: Case Report and Review

**DOI:** 10.7759/cureus.4895

**Published:** 2019-06-13

**Authors:** Ricardo Correa, Christian Ortega-Loubon, Luis E. Zapata-Castro, Blas Armién, Carlos Culquichicón

**Affiliations:** 1 Endocrinology, Diabetes and Metabolism, University of Arizona College of Medicine-Phoenix, Phoenix, USA; 2 Cardiac Surgery, Clinic University Hospital of Valladolid, Valladolid, ESP; 3 Emerge Piura, Emerging Diseases and Climate Change Research Unit, School of Health Sciences, Universidad Nacional de Piura, Piura, PER; 4 Vectors and Transmissible Diseases, Gorgas Memorial Institute of Health Studies, Panama City, PAN; 5 Emerge Piura, Emerging Diseases and Climate Change Research Unit, Universidad Nacional de Piura, Piura, PER

**Keywords:** dengue with hemorrhagic manifestations, classic dengue, acute pancreatitis, abdominal pain

## Abstract

Dengue fever is an acute viral disease transmitted by arthropods, which has become a major public health problem, especially in tropical and subtropical areas. We found 17 reported cases of dengue complicated with pancreatitis in the world literature, 13 cases were found in Asia, one in Europe, and two in Latin America. This is the first and an unusual case of dengue with hemorrhagic manifestations complicated with acute pancreatitis in Panama.

A 37-year-old woman with an unknown past medical history presented to Santo Tomas Hospital (Panama City, Panama) with sudden abdominal pain in the epigastrium and left hypochondrium, described as a burning-like pain radiating towards the back. Five days before the admission, the patient had fever, chills, headache, retro-orbital pain, dizziness, arthralgia, loss of appetite, and fatigue. In the emergency room, a diagnosis of acute pancreatitis was made and the patient was hospitalized. Physical examination showed bleeding gums, and laboratory tests show leukopenia and thrombocytopenia, which suggested an infection caused by the dengue virus. The dengue infection was confirmed using quantitative polymerase chain reaction (qPCR) and enzyme-linked immunosorbent assay (ELISA). The patient received supportive treatment and the symptoms resolved. She was discharged on the ninth day of hospitalization without any sequela. The final diagnosis was hemorrhagic dengue complicated by acute pancreatitis.

## Introduction

Dengue fever is an acute disease transmitted by arthropods with a large geographical distribution [1]. It mainly affects the Pacific Islands, Asia, and the Americas and has recently become a major global public health concern [1]. It is caused by the dengue pathogen, which is an enveloped ribonucleic (RNA) virus, from the Flaviviridae family [1]. The infection caused by the dengue virus causes a wide spectrum of diseases that can be life-threatening, such as dengue with hemorrhagic manifestations and severe dengue triggering dengue shock syndrome [1]. During the last 25 years, Panama has reported multiple outbreaks; they have been affected in a total of 68,175 people [2]. Nowadays, up to 2018 Fall, Panama has reported the circulation of DENV-1 and DEN-2, with a cumulative incidence rate of 128.57 per 100,000 inhabitants and a case fatality rate of 0.057, being a risk to public health [3].

Apart from the well-known typical manifestations, other unusual manifestations have been reported, such as abdominal pain (41.3%), hepatitis (40.6%), renal failure (8%), cholecystitis (6.66%), conduction abnormalities of heart (6%), myocarditis (3.33%), acute pancreatitis (1.33%), respiratory distress (1.33%), myositis (0.66%), and encephalitis (0.66%) [4]. These abnormal conditions make the diagnosis and treatment very challenging and complicate the patient’s survival [4]. 

After a deep browse through of Scopus, MEDLINE, Sci-E, SciELO, and BVS, we found 17 reported cases of dengue complicated with pancreatitis in the worldwide literature, of which 13 cases were found in Asia, one in Europe and two in Latin America [5-12]. We report the first and an unusual case of dengue with hemorrhagic manifestations complicated with acute pancreatitis in Panama.

## Case presentation

A 37-year-old female patient, with an unknown past medical history, presented to the emergency room (ER) at Santo Tomas Hospital (Panama City, Panama), with acute-onset burning abdominal pain in the epigastrium and left hypochondrium that radiates towards the back. The pain was so intense that, sometimes, it was difficult for her to breath. Five days before the admission, the patient had a fever of over 39.1°C, chills, headache, retro-orbital pain, dizziness, arthralgia, loss of appetite, and fatigue. To control the previous symptoms, she self-prescribed acetaminophen.

Physical examination revealed a blood pressure of 110/80 mmHg, a heart rate of 72 beats/min, a respiratory rate of 20 breaths/min, anicteric sclera, gingivorrhagia, abdomen with pain on palpation in the epigastrium, no organomegaly, no rebound tenderness, no guarding, negative Murphy’s sign, and no evidence of spontaneous bleeding or petechial over the body. Lab tests done on the day of admission: hemoglobin 13.9 g/dL, hematocrit 41.9%, leukocytes 3,700/mm3, 88 platelets x 109/L, amylase 225 U/L, lipase 917 U/L, alanine aminotransferase (AST) 138 U/L, aspartate aminotransferase (ALT) 105 U/L, lactate dehydrogenase (LDH) 1397U/L, and albumin 2.5 g. Also, the bilirubin levels, creatinine, and C-reactive protein were within normal limits. Chest X-ray did not show any infiltrate or pleural effusion. An abdominal radiograph showed the normal distribution of the intestinal area; renal shadows and psoas were normal. Given the clinical characteristics and lab alterations suggesting hemorrhagic dengue and acute pancreatitis, the decision was made to hospitalize the patient.

Her abdominal pain continued, then an abdominal ultrasound (USG) was performed, demonstrating the normal size of the liver and pancreas; biliary pathways were normal; the gallbladder was 4 mm thick without edema and without visible lytes. Computed tomography (CT) was indicated but was not done. On the second day of admission, the patient developed a diffuse erythematous rash associated with pruritus, so treatment with an antihistamine was started. Human immunodeficiency virus (HIV) and venereal disease research laboratory (VDRL) tests were negative. Cytomegalovirus: only immunoglobulin G (IgG) was positive. On the third day, the patient’s abdominal pain quickly resolved and an oral regimen was started.

The dengue outbreak experienced in the country during those times, along with the clinical characteristics experienced by the patient, indicated that dengue was the cause of the patient’s illness. Eight days after the symptoms had first appeared, a serum sample was sent to the Gorgas Memorial Institute of Health Studies for a serological test for dengue.

After supportive care with intravenous fluids for three days, the abdominal pain and fever resolved and the complete blood count (CBC) and basic metabolic panel (BMP) corrected. The patient’s clinical condition became stable and she was discharged. One week after discharge, the Gorgas Commemorative Institute for Health Studies reported immunoglobulin M (IgM), enzyme-linked immunosorbent assay (ELISA), and qPCR positive for dengue. The case was concluded as dengue with hemorrhagic manifestations complicated by acute pancreatitis.

## Discussion

Some tropical viral infections can rarely cause pancreatitis, however, through the several series, they could cause up to 10% of pancreatitis cases [13]. Some examples of this include hepatitis virus (0%-0.1%), enterovirus, cytomegalovirus, Epstein-Barr virus (nine cases reported so far), varicella-zoster virus (three cases reported so far), rubella virus, Ascaris, mycoplasma, Campylobacter, Mycobacterium avium, and dengue virus (17 cases in the in the worldwide literature) [14]. The physiopathology behind the acute pancreatitis in this infection is not well understood although there are two hypotheses that had better explain this process: (1) the pancreatic compromise of the biliary pathway and (2) the small hemorrhages in the peritoneal cavity [15]. The first hypothesis suggests that the viral infection causes an autoimmune response to pancreatic islet cells, thus edema develops in the Vater's ampulla and obstructs the exit of pancreatic fluids [15]. The second hypothesis suggests that acute pancreatitis is caused by the direct inflammation and destruction of the pancreatic acinar by the same virus; this is mainly evidenced in dengue shock syndrome (grades III and IV of dengue hemorrhagic fever (DHF)) [15].

During a deep browse in the literature, we identified 15 articles reporting 17 patients with pancreatitis due to dengue fever. In summary, 76.5% (n=13) of cases were reported from Asia, 11.76% (n=2) from Latin America, and 5.9% (n=1) from Europe. Taiwan and Bangladesh were the countries with the highest reported cases, with 29.4% and 11.8%, respectively. The most prevalent serotype was DEN-1 in 64.71% of the cases and 35.3% of the cases did not report the serotype. The most frequent symptoms were fever (52.9%, nine cases), abdominal pain (47.1%, eight cases), vomiting (47.1%, eight cases), body pain (35.3%, six cases), chills (23.5%, four cases), and dyspnea (17.7%, three cases), and most of them were males (47.1%, eight cases). Regarding laboratory tests, 11.8% of the cases reported hypocalcemia, 41.2% had hyperlipasemia, 41.2% had hyperamylasemia, 35.3% had thrombocytopenia, 35.3% had dengue IgM and IgG positive detection, 11.8% had an NS1 positive antigen, 5.9% had edema of the thick gallbladder wall, 64.7% had low hematocrit, 5.9% had high pleural fluid amylase, 35.3% were dengue virus IgM positive and negative IgG, 5.8% of tests were of renal-derived function, 5.9% had lymphopenia, and 5.9% had hemoconcentration. About the pancreas examinations, 94.1% of the cases were diagnosed with pancreatitis with a CT scan and 5.9% reported hemorrhagic pancreatitis. The final diagnoses of the reported cases corresponded 64.71% to DHF grade I, 17.7% to DHF grade II, and 5.9% to DHF grade IV. In 70.6% of the cases, the patient was discharged, and 11.8% of them died.

In fact, detection of the serological virus can confirm the diagnosis of DH; the disadvantage is that the results are often obtained in late stages [16]. Another diagnostic tool is the transabdominal ultrasound, which should be used for screening patients with acute pancreatitis despite the low quality of evidence that supports this recommendation [16]. Indeed, this complex clinical scenario encourages the use of additional resources that are available timely. One of them is to evaluate the thickness of the gallbladder by ultrasound, essentially to screen the diagnosis of DHF [17]. A thickness of the gallbladder wall greater than 3 mm has high sensitivity (87%) and low specificity (48%) to screen DHF [18]. This pattern can also be used to predict the conditions of the disease and the recovery or complications of the patient [17]. Our patient had a gallbladder wall thickness of 4 mm, which supports the diagnosis of DHF. Moreover, the current trends of innovative diagnostic tests show that the determination of urinary histamine is a reliable marker to diagnose severe dengue cases [19]. The histamine plays an important role in the leakage of intravascular fluid to the various serous spaces, which produces hypovolemia and shock in severe dengue [19].

We should understand the results under the scope of a reliable clinical, and laboratory approach with expertise in tropical medicine from the Gorgas Institute mentor’s team, thus overcoming the lack of a CT scan for a confirmatory diagnosis. Moreover, following a solid methodology for clinical case report [20] brings confidence to the findings.

## Conclusions

In the context of febrile patients with compatible symptoms of dengue, the presence of acute and intense abdominal pain and vomiting should indicate acute pancreatitis. In all reported cases, the hyperlipasemia, hyperamylasemia, and dengue IgM and IgG positivity were the tests that best detected dengue complicated with acute pancreatitis. A gallbladder wall thickness of greater than 3 mm could be considered a complementary test for screening dengue hemorrhagic fever.
